# Efficacy and safety of angiogenesis inhibitors combined with poly ADP ribose polymerase inhibitors in the maintenance treatment of advanced ovarian cancer: a meta-analysis

**DOI:** 10.3389/fonc.2024.1477105

**Published:** 2024-11-18

**Authors:** Renming Huang, Feng Ji, Leyi Huang, Yueying Qin, Zhiyu Liang, Miaoyan Huang, Chunyan Li, Jian Ban

**Affiliations:** The First Affiliated Hospital of Guangxi University of Science and Technology, Guangxi University of Science and Technology, Liuzhou, China

**Keywords:** ovarian cancer, angiogenesis inhibitor, poly ADP ribose polymerase inhibitor, PARPi, objective response rate, progression-free survival, overall survival, meta-analysis

## Abstract

**Introduction:**

This meta-analysis was performed to evaluate the efficacy and safety of angiogenesis inhibitors (Ais) combined with poly ADP ribose polymerase inhibitors (PARPi) in the maintenance treatment of advanced ovarian cancer (OC).

**Materials and methods:**

A systematic search was conducted in four databases (Pubmed, Embase, Web of Science, and Cochrane) for articles published from the inception of the databases until January 15, 2024. The focus of the search was on articles investigating the combination of Ais with PARPi in the maintenance treatment of ovarian cancer. Meta-analyses were conducted to assess the objective response rate (ORR), progression-free survival (PFS), overall survival (OS), and the risk of Grade ≥ 3 adverse events (Grade≥ 3 AEs).

**Results:**

Totally nine studies were included for meta-analysis. The overall pooled ORR of Ais combined with PARPi was 57% (95% CI, 35% to 77%). Subgroup analyses showed that the ORR for patients with platinum-resistant recurrent ovarian cancer, platinum-sensitive recurrent ovarian cancer and newly diagnosed advanced ovarian cancer were 30% (95% CI, 12% to 52%), 70% (95% CI, 61% to 78%) and 59% (95% CI, 55% to 63%), respectively. The median PFS was 5.8 months (95% CI, 5.3 to 7.1), 12.4 months (95% CI, 10.6 to 13.2) and 22.4 months (95% CI, 21.5 to 24.2), respectively. The median OS was 15.5 months (95% CI, 12.3 to 24.8), 40.8 months (95% CI, 33.4 to 45.2) and 56.3 months (95% CI, 49.0 to 62.0), respectively. The rate Grade≥ 3 TRAEs rate was found to be 0.22 (95% CI, 0.13 to 0.33).

**Conclusions:**

Our results confirmed that PARPi plus Ais was a feasible and safe option for the maintenance treatment of advanced ovarian cancer. The combination therapy should be recommended as the first-line maintenance treatment for patients with advanced ovarian cancer. PARPi plus Ais yielded more favorable oncological prognosis for patients with platinum-sensitive recurrent ovarian cancer, compared to patients with platinum-resistant recurrent ovarian cancer.

**Systematic review registration:**

https://www.crd.york.ac.uk/prospero/display_record.php?ID=CRD42024543590, identifier CRD42024543590.

## 1 Introduction

Ovarian cancer (OC) ranks as the fifth most common cause of mortality due to cancer in women, with a survival rate of fewer than 50% over a period of five years (1). In the majority of instances, the main approach to treating ovarian cancer involves combining cytoreductive surgery with systemic chemotherapy (2, 3). Although the majority of patients experience a complete response to primary treatment, most will eventually experience a recurrence (4). The condition is associated with a pessimistic prognosis, as the average length of survival is less than 12 months (5).

Targeted therapy is now widely recognized as a successful treatment for various forms of cancers (6). Targeted therapy, such as anti-angiogenesis inhibitors (Ais) and poly ADP ribose polymerase inhibitors (PARPi), as an adjuvant therapy alongside chemotherapy and radiation therapy, can significantly improve treatment outcomes (7). Three PARPis, namely olaparib, niraparib, and rucaparib, have been granted approval by the US Food and Drug Administration (FDA) for use as maintenance therapy in OC patients (8). Olaparib, niraparib, and rucaparib have been approved for maintenance and third-line treatment, respectively, in Europe for platinum-sensitive, relapsed, BRCA1/2 mutated ovarian cancer after a complete or partial response (CR/PR) to platinum-based chemotherapy (9). Maintenance treatment with olaparib plus bevacizumab should be explored for patients with recently diagnosed advanced ovarian cancer, regardless of clinical risk, according to the PAPLA-1 and OVARIO trials (8). In the PAOLA-1 study, the median progression-free survival (PFS) reported by investigators was 22.1 months for olaparib plus bevacizumab and 16.6 months for placebo plus bevacizumab (10). The combination of niraparib with bevacizumab for maintenance therapy resulted in a primary endpoint of 18-month PFS rate of 62% in the intention-to-treat (ITT) population, with a median PFS of 19.6 months (11). Due to the notable clinical advantages of combining Ais with PARPi observed in ovarian cancer patients, there has been a rise in the number of clinical trials investigating the safety and effectiveness of this combination for the maintenance treatment of advanced ovarian cancer.

In the present study, we aimed to systematically assess the available evidence in the literature regarding the efficacy and safety of maintenance Ais combined with PARPi for patients with advanced ovarian cancer.

## 2 Material and methods

### 2.1 Search strategy

The current meta-analysis was performed in accordance with the Preferred Reporting Items for Systematic Reviews and Meta-Analyses (PRISMA) 2020 standards. This study has been registered at PROSPERO with a registration number of CRD42024543590. A systematic search was conducted in four databases, namely PubMed, Embase, Web of Science, and the Cochrane Library, to retrieve literature published until January 15, 2024. The search method used the following terms: “ovarian cancer,” “PARP inhibitor,” “angiogenesis inhibitors,” and “randomized controlled trial.” We also conducted a thorough manual examination of the bibliographies of the identified papers, as well as pertinent reviews and meta-analyses, in order to uncover any new research that fit the criteria for inclusion. **Supplementary Material 1** provided a comprehensive overview of the search record.

### 2.2 Inclusion and exclusion criteria

Inclusion criteria: (1) patients diagnosed with advanced ovarian cancer, fallopian tube cancer, or primary peritoneal cancer; (2) at least one cohort of patients was administered maintenance Ais combined with PARPi; (3) At least one of the following outcomes was reported: ORR, PFS, OS, Grade ≥ 3 AEs; (4) Study types: randomized controlled studies, single-arm trials.

Exclusion criteria: (1) other types of articles, such as case reports, publications, letters, reviews, meta-analyses, editorials, pharmacological intervention, animal studies and protocols; (2) other diseases; (3) irrelevant studies; (4) failed to extract data; (5) duplicate patient cohort.

### 2.3 Selection of studies

The process of literature selection, which involved removing duplicate entries, was conducted using EndNote (Version 21; Clarivate Analytics). The initial search was undertaken by two independent reviewers. The duplicate records are eliminated, the titles and abstracts were assessed to ascertain their relevance, and each study was categorized as either included or excluded. We arrived at a resolution through the consensus. If the parties were unable to reach an agreement, a third reviewer acted as a mediator.

### 2.4 Data extraction

Two autonomous reviewers conducted a thorough examination of the title and abstract, followed by a comprehensive reading of the entire text. The discrepancies were resolved through consultation with a third investigator. The collected data consist of the first author’s name, year of publication, study area, trail ID, study design, sample size, intervention, age of participants, trial phase, study period, median follow-up duration, ORR, Grade ≥ 3 AEs, Kaplan-Meier curves for OS and Kaplan-Meier curves for PFS.

### 2.5 Risk of bias assessment

Two unbiased reviewers evaluated the quality assessment of the included studies. We utilized the modified Jadad scale (12) as well as Cochrane Collaboration risk of bias assessment tool (ROB) to assess the quality of randomized controlled trials in this analysis. The single-arm trials were evaluated using the methodological index for non-randomized studies (MINORS) (13).

### 2.6 Statistical analysis

The selection of duplicates of studies included was conducted using EndNote (Version 21; Clarivate Analytics). All analyses were performed using Stata 16.0. The “meta” package and IPDformKM package were utilized in the analysis. GetData Graph Digitizer software was used to extract data from articles containing Kaplan-Meier curves, and individual data were reconstructed using the IPDformKM package. The established method by Guyot et al. was used to reconstruct individual patient-level data (14). Continuous variables were compared using the weighted mean difference (WMD) with a 95% confidence interval (CI). Relative ratio (RR) with 95% CI was used to compare binary variables. The medians and interquartile ranges of continuous data were converted to means and standard deviations. Statistical heterogeneity between included studies was calculated using the Cochrane ‘Sq test and the I^2^ index (I^2^ >50% indicating significant heterogeneity). When there was high heterogeneity among studies, the random effects model was adopted, otherwise the fixed effects model was adopted (15). P values < 0.05 was considered statistically significant. Finally, a sensitivity analysis was performed to determine the impact of individual studies on the aggregated results and to test the reliability of the results.

## 3 Results

### 3.1 Search results

**Figure 1:**
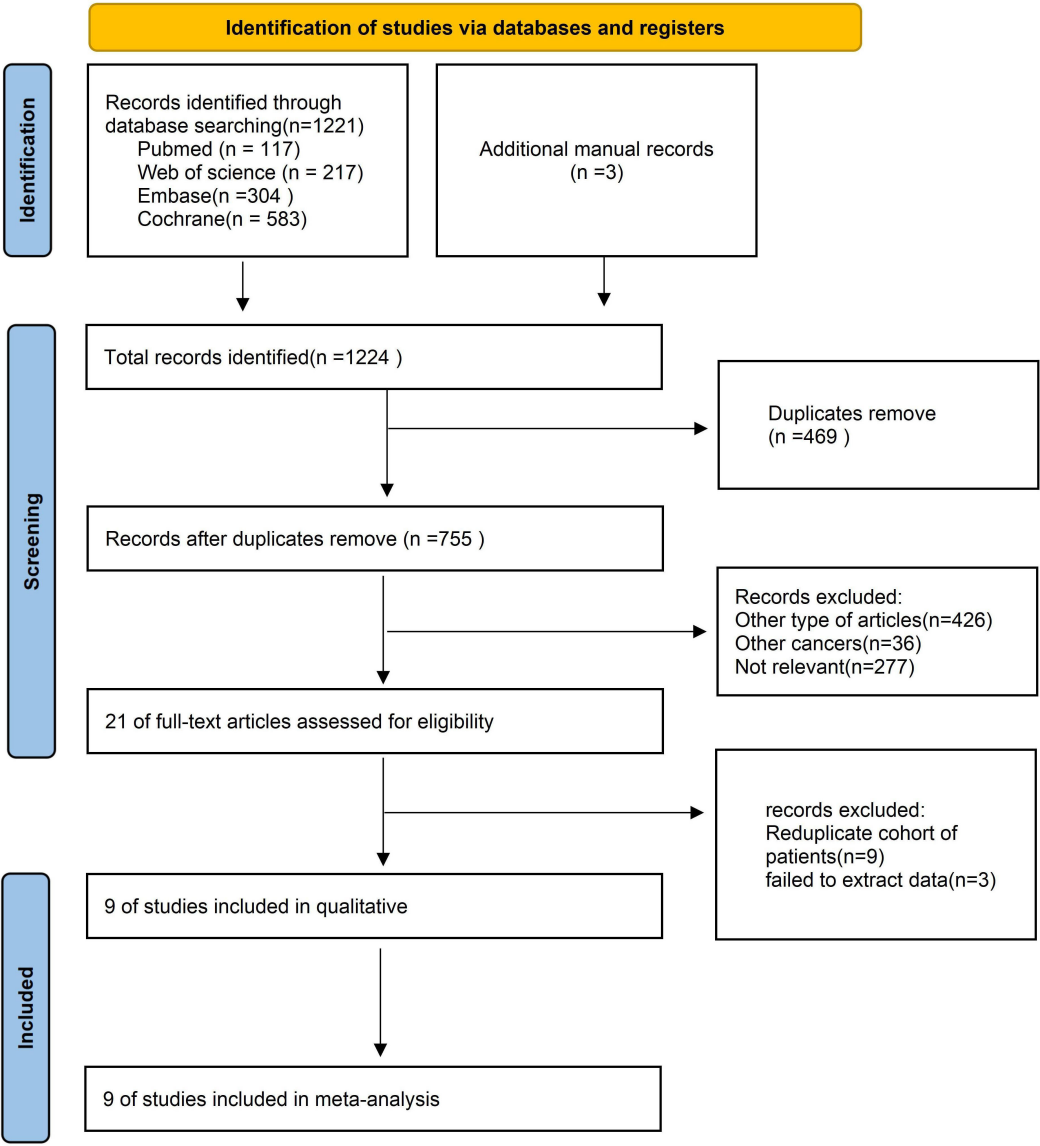
Flow chart of literature search strategies.

**Figure 1** illustrates the process of selecting and incorporating literature. We initially discovered a grand total of 1,224 studies. After eliminating unnecessary research, a total of 755 papers were kept. After assessing the titles and abstracts, a grand total of 24 publications were deemed inappropriate and thus excluded. Following a thorough examination of the entire text, a total of nine studies were included for inclusion in this meta-analysis.

### 3.2 Patient characteristics and quality assessment

**Figure 2:**
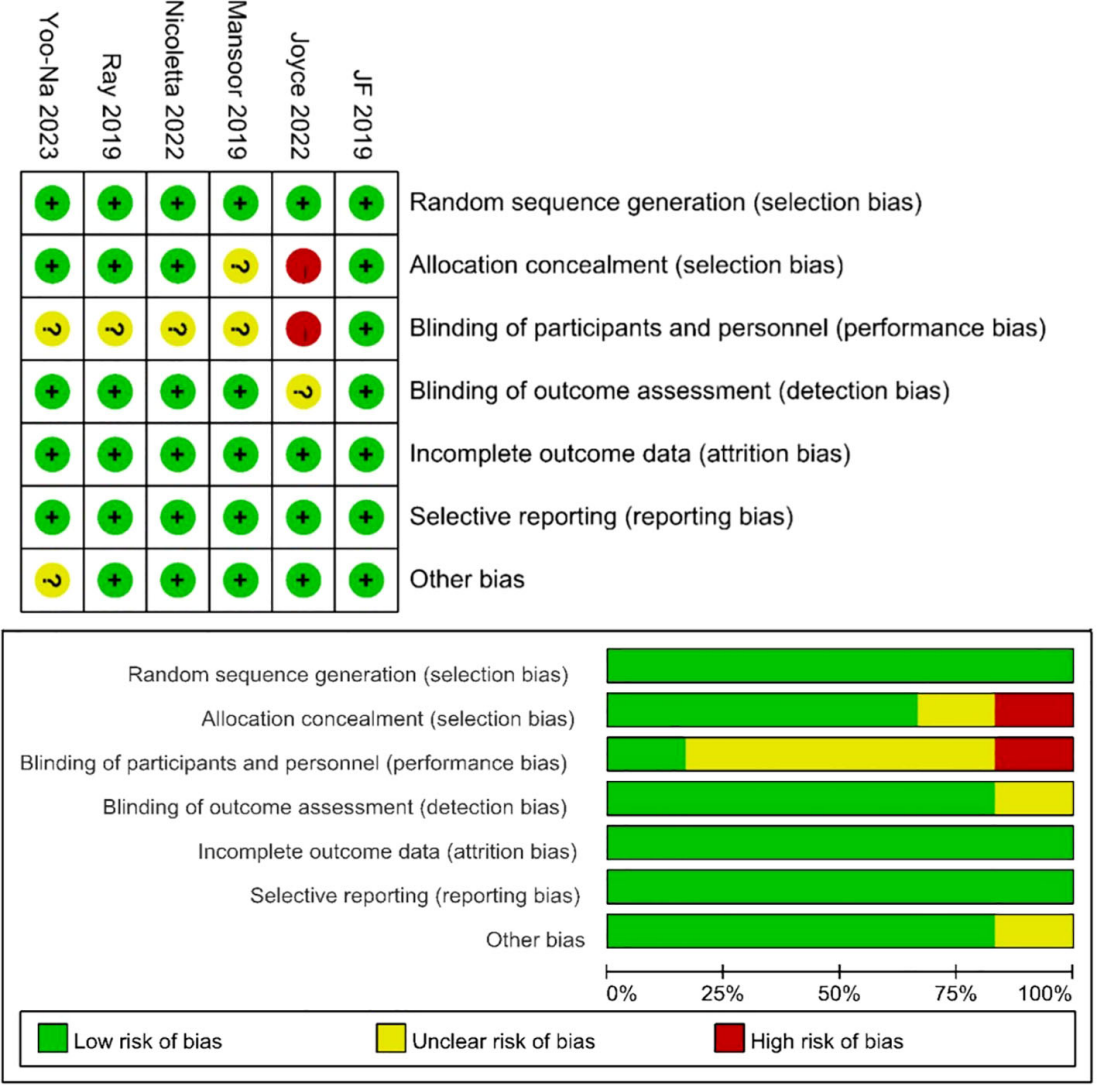
Risk of bias assessment for the RCT studies.

**Table 1** presents detailed data on patient characteristics and quality assessment. A total of 9 articles were included in this article, including 6 RCTs (10, 16–20) and 3 single-arm trials (11, 21, 22), with a total of 1074 patients diagnosed with diagnosed with advanced ovarian cancer. The authors, registration ID, year, region, study design, study arm, patients, age, and median follow-up for each study are shown in **Table 1**. The meta-analysis focused exclusively on the data of patients who received Ais combined with PARPi. A subgroup analysis was conducted to address inconsistencies in the patient inclusion criteria across various trials, encompassing platinum-resistant recurrent ovarian cancer (17, 18, 21, 22), platinum-sensitive recurrent ovarian cancer (16, 19, 20), and newly diagnosed advanced ovarian cancer (10, 11). The specific regimens and doses in each trial were provided in **Table 1**. Six trials used Olaparib (10, 16–18, 20, 21) and three used Niraparib (11, 19, 22). We employed the modified Jadad scale to evaluate the quality of the RCT literature for quality assessment. The single-arm studies were evaluated using the MINORS methodology. All trials were considered to be of high quality (**Table 1**). **Figure 2** presents a concise overview of the risk of bias assessment results for RCTs using ROB.

**Table 1:** Characteristics of included studies and patients.

Author	Registration ID	Region	Study design	Subgroup	Regimens	Sample	Age (median, years)	Median follow-up (months)	Quality
Jung-Min 2022 (21)	NCT02889900	USA	single-arm	A	Cediranib30mgqd+Olaparib200mgbid	60	64.5	NA	14
Guochen 2022 (22)	NCT04376073	China	single-arm	A	Anlotinib10mgqd+Niraparib200-300mgqd	40	54	15.4	13
Nicoletta 2022 (17)	NCT03314740	Italy	RCT	A	Cediranib20mgqd+Olaparib300mgbid	41	64.2	29.7	5
Yoo-Na 2023 (18)	NCT03699449	South Korea	RCT	A	Cediranib30mgqd+Olaparib200mgbid	16	NA	NA	4
Mansoor 2019 (19)	NCT02354131	Europe、USA	RCT	B	Bevacizumab15mg/kgq3w+Niraparib300mgbid	48	67	16.9	4
Joyce 2022 (16)	NCT02446600	USA	RCT	B	Cediranib30mgqd+Olaparib200mgbid	183	NA	NA	4
JF 2019 (20)	NCT01116648	USA	RCT	B	Cediranib30mgqd+Olaparib200mgbid	44	NA	46	4
Ray 2019 (10)	NCT02477644	France	RCT	C	Bevacizumab15mg/kgq3w+Olaparib300mgbid	537	61.0	22.9	5
Melissa 2022 (11)	NCT03326193	USA	single-arm	C	Bevacizumab15mg/kgq3w+Niraparib200mgbid	105	60	28.7	14

Subgroup: A, platinum-resistant recurrent ovarian cancer; B, platinum-sensitive recurrent ovarian cancer; C, newly diagnosed advanced ovarian cancer

### 3.3 ORR

**Figure 3:**
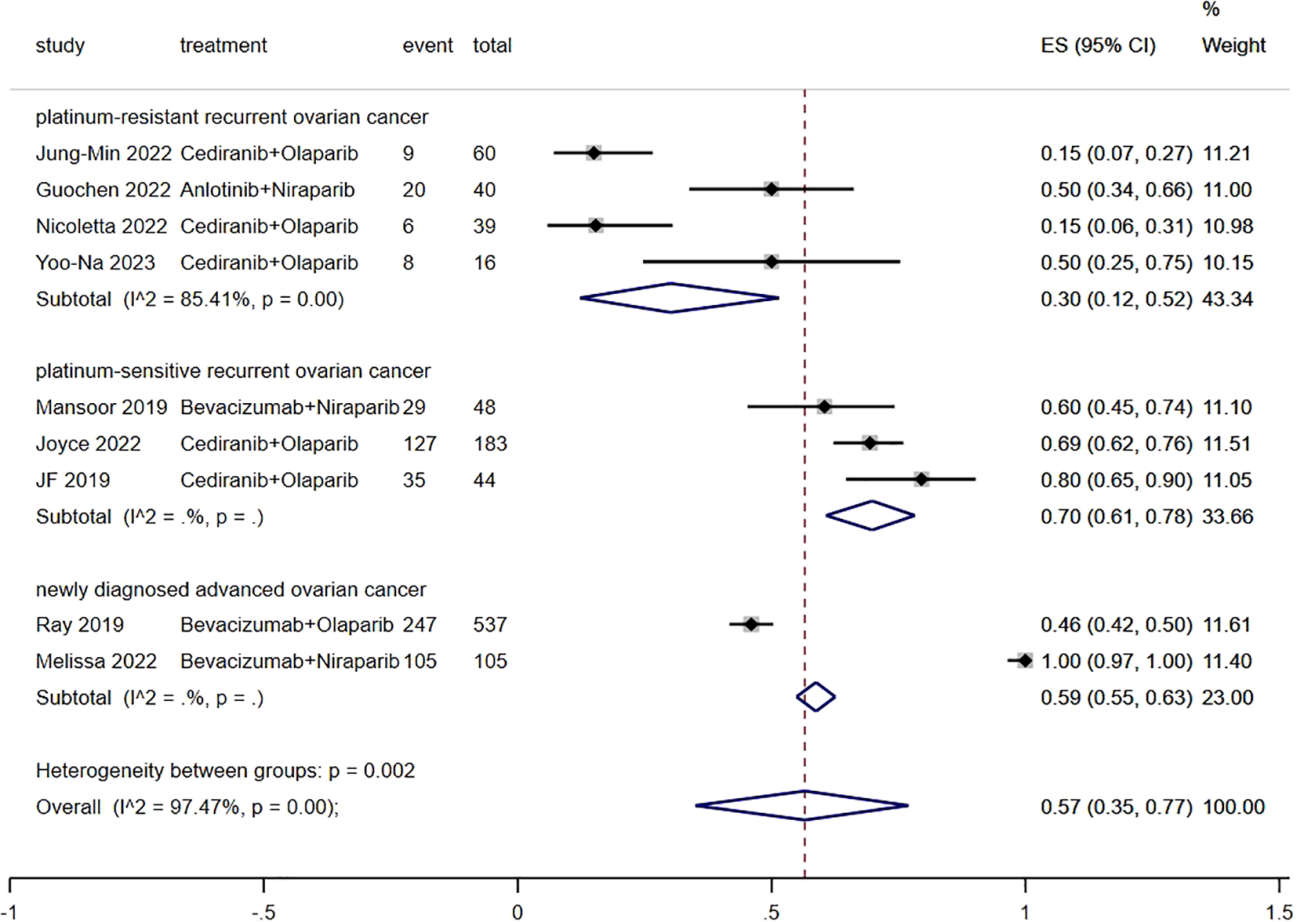
Forest plot of the meta-analysis for ORR.

**Figure 3** presents the result of ORR. The ORR of patients with advanced ovarian cancer who received Ais combined with PARPi was 57% (95% CI, 35% to 77%). In subgroup analysis, the ORR of patients with platinum-resistant recurrent ovarian cancer, patients with platinum-sensitive recurrent ovarian cancer and patients with newly diagnosed advanced ovarian cancer was 30% (95% CI, 12% to 52%), 70% (95% CI, 61% to 78%) and 59% (95% CI, 55% to 63%), respectively.

### 3.4 PFS

**Figure 4:**
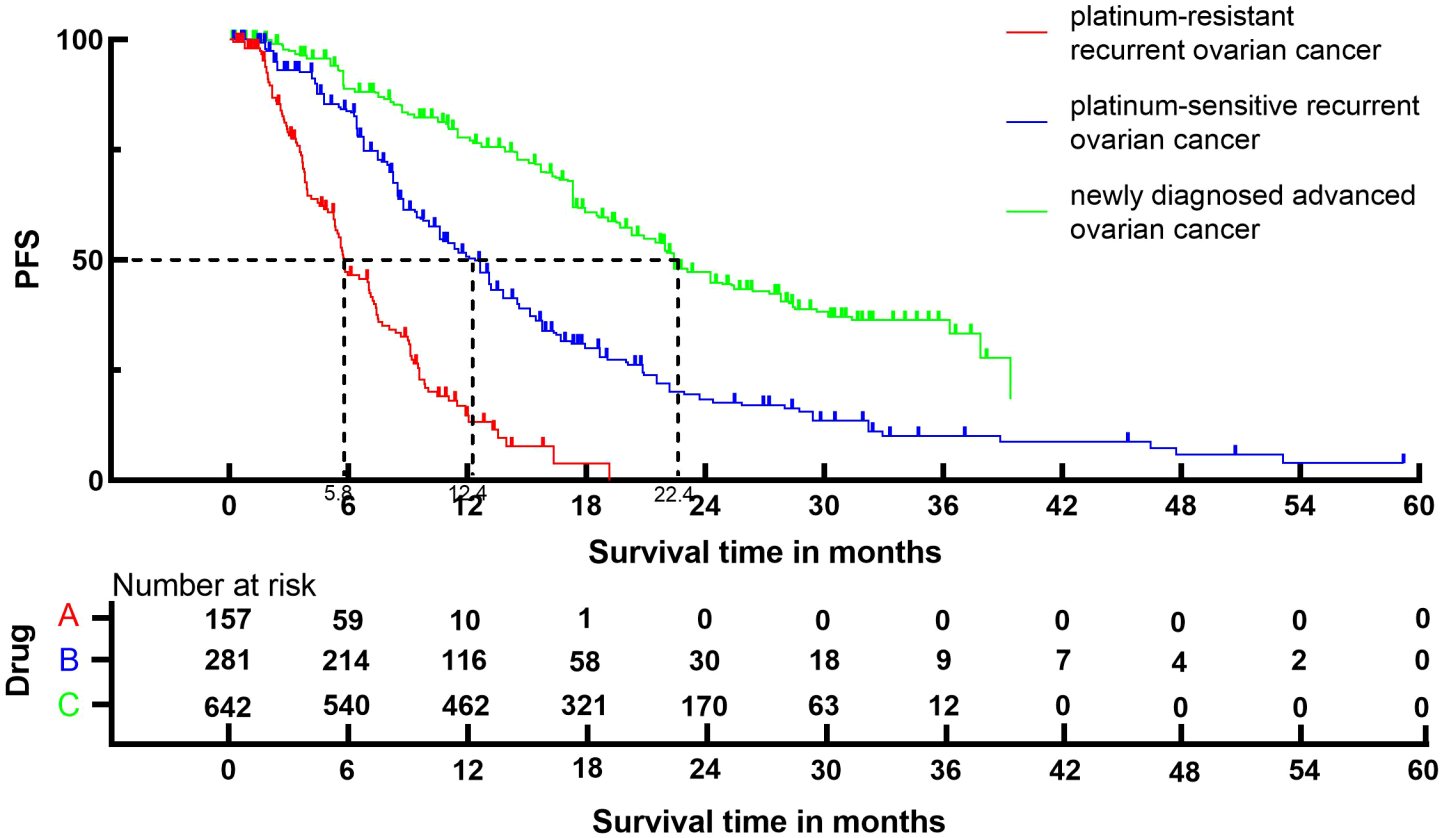
Kaplan-Meier curves for PFS.

Following the reconstruction of the cohort, we conducted an additional evaluation of PFS using a Kaplan-Meier curve (**Figure 4**). The median PFS of patients with platinum-resistant recurrent ovarian cancer, patients with platinum-sensitive recurrent ovarian cancer and patients with newly diagnosed advanced ovarian cancer were 5.8 (95% CI, 5.3 to 7.1) months, 12.4 (95% CI, 10.6 to 13.2) months and 22.4 (95% CI, 21.5 to 24.2) months, respectively.

### 3.4 OS

**Figure 5:**
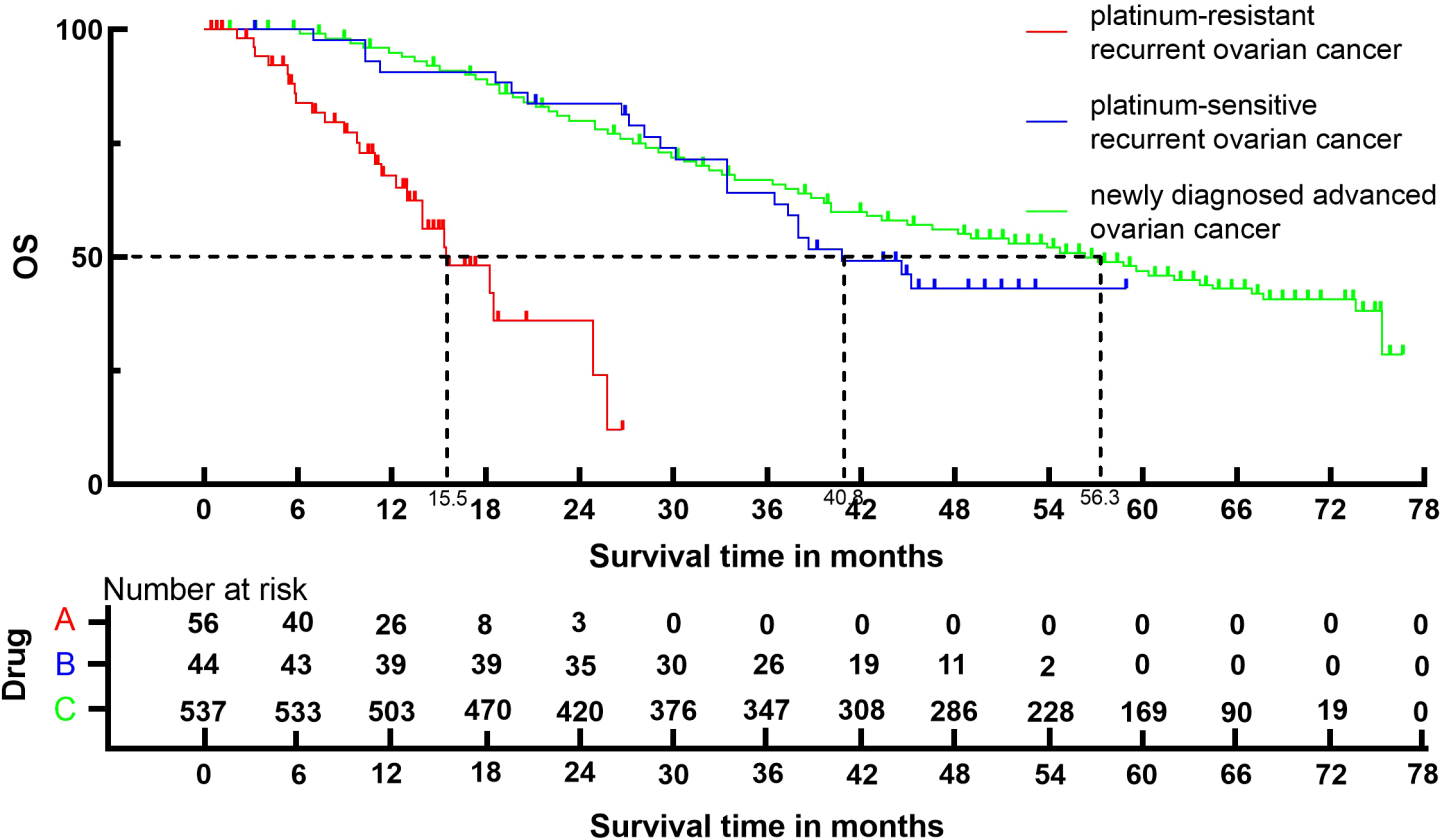
Kaplan-Meier curves for OS.

Following the reconstruction of the cohort, we conducted an additional evaluation of OS using a Kaplan-Meier curve (**Figure 5**). The median OS of patients with platinum-resistant recurrent ovarian cancer, patients with platinum-sensitive recurrent ovarian cancer and patients with newly diagnosed advanced ovarian cancer were 15.5 (95% CI, 12.3 to 24.8) months, 40.8 (95% CI, 33.4 to 45.2) months and 56.3 (95% CI, 49.0 to 62.0) months, respectively.

### 3.5 Grade≥ 3 TRAEs rate

**Figure 6:**
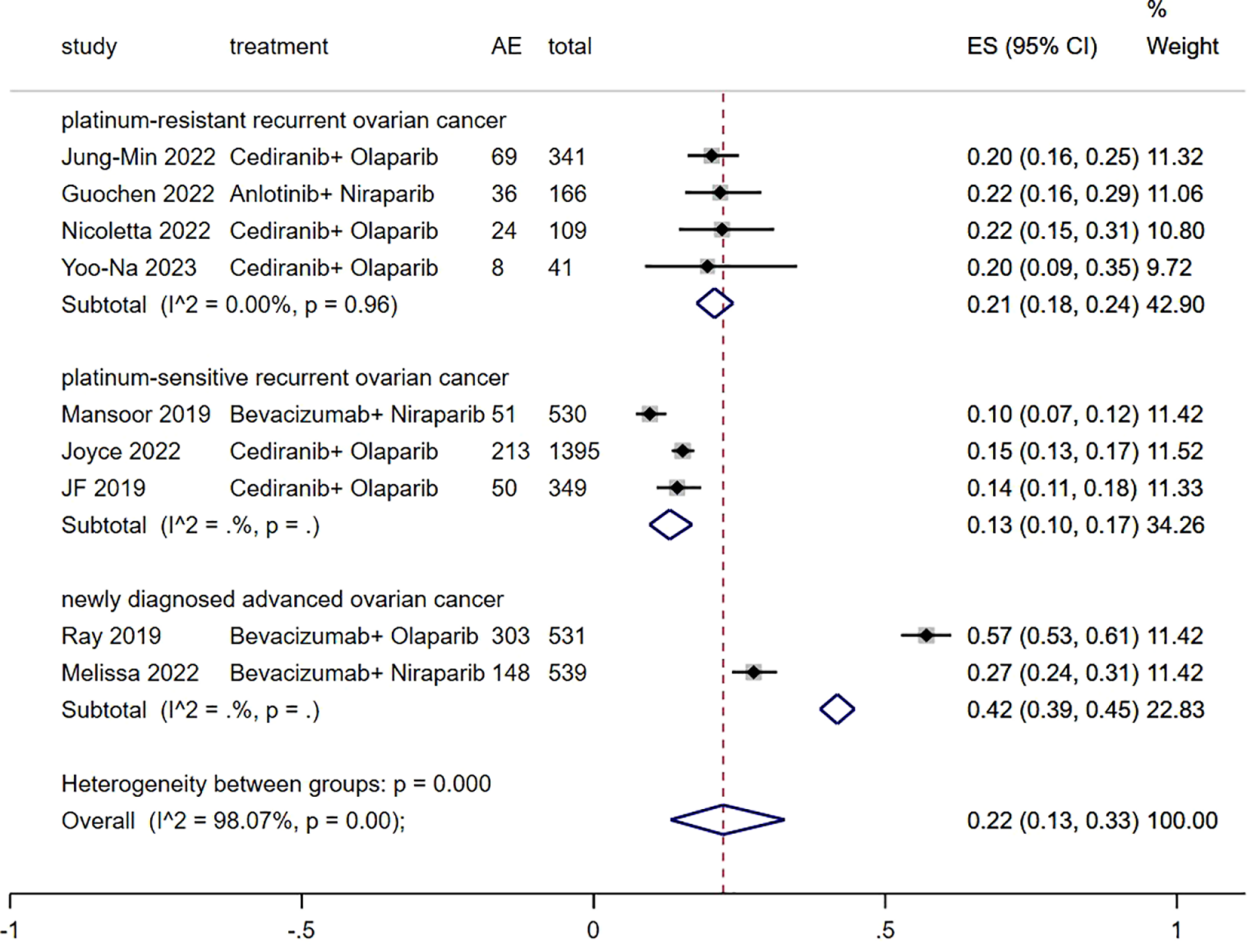
Forest plot of the meta-analysis for Grade≥ 3 AEs rate.

The Grade≥ 3 TRAEs rate was found to be 0.22 (95% CI, 0.13 to 0.33) (**Figure 6**) among patients with advanced ovarian cancer who received Ais combined with PARPi. In subgroup analysis, the Grade≥ 3 TRAEs rate in patients with platinum-resistant recurrent ovarian cancer, patients with platinum-sensitive recurrent ovarian cancer and patients with newly diagnosed advanced ovarian cancer was 0.21 (95% CI, 0.18 to 0.24), 0.13 (95% CI, 0.10 to 0.17), and 0.42 (95% CI, 0.39 to 0.45) respectively.

## 4 Discussion

There has been an increase in the number of clinical trials studying the safety and effectiveness of maintenance combining Ais with PARPi for treating advanced ovarian cancer, due to the significant therapeutic benefits found in patients. In this study, we performed the first systematic review and meta-analysis to thoroughly evaluate the effectiveness and safety of maintenance combining Ais with PARPi for patients with advanced ovarian cancer.

The findings from our study revealed that patients with advanced ovarian cancer who were administered Ais plus PARPi had an ORR of 57%, indicating the favorable antitumor efficacy of the combined treatment. A subgroup analysis was performed to address discrepancies in the patient inclusion criteria among different trials, including patients with platinum-resistant recurrent ovarian cancer, platinum-sensitive recurrent ovarian cancer, and newly diagnosed advanced ovarian cancer. The ORR of patients with platinum-resistant recurrent ovarian cancer, patients with platinum-sensitive recurrent ovarian cancer and patients with newly diagnosed advanced ovarian cancer was 30%, 70% and 59%, respectively. This finding suggests that the resistance to Ais combined with PARPi may be linked to resistance to platinum. The outcomes of OS and PFS demonstrated that the combination of Ais and PARPi yielded more favorable oncological prognosis for patients with platinum-sensitive recurrent ovarian cancer, as opposed to patients with platinum-resistant recurrent ovarian cancer. Specifically, the notable advantage of Ais and PARPi for patients with advanced ovarian cancer was evident in terms of PFS and OS, confirming that the combination therapy should be recommended as the first-line therapy for patients with advanced ovarian cancer.

Ais play a crucial role in malignancy treatment (23) by disrupting the formation of new blood vessels, which tumor cells require for growth and metastasis (24). Specifically, Cediranib acts as a potent inhibitor of vascular endothelial growth factor receptors (VEGFRs), effectively blocking endothelial cell proliferation and survival (25). Bevacizumab, a monoclonal antibody, directly neutralizes VEGF, preventing its interaction with receptors on the endothelial cell surface (26). Anlotinib, a multi-targeted kinase inhibitor, not only inhibits VEGFR but also acts on other pathways like PDGFR and FGFR, reinforcing its anti-angiogenic effects in advanced ovarian cancer treatment (27). Ais or PARPi exhibit substantial efficacy as monotherapy for recurrent ovarian cancer, but, prolonged usage of a single agent can lead to the development of drug resistance (9). Previous research suggests that Ais plus PARP inhibition may boost anticancer efficacy (28, 29). Studies have demonstrated that antiangiogenic drugs have multiple effects on homologous recombination deficiency (HRD), including reducing angiogenesis, creating hypoxia in tumor microenvironment, and downregulating BRCA1/2 and RAD51, two of the most important components in HRD (29–31). Bevacizumab is linked to an elevated occurrence of hypoxia-induced HRD deficiency in tumor cells (32). Cells exhibiting heightened HRD may display more vulnerability to PARPi, whereas Ais have the ability to bind with PARPi, resulting in mutually reinforcing anticancer effects (28). Homologous recombination repair deficiency is a frequent feature of high-grade serous ovarian, fallopian tube and peritoneal carcinoma (HGSC) and is associated with sensitivity to PARP inhibitor (33). Additionally, PARPi may possibly have a role in the process of angiogenesis. Although vascular growth factor is present, the deletion of PARP1 in mice resulted in a decrease in angiogenesis, indicating a possible antiangiogenic action of PARPi (34). The upregulation of PARPi in human epithelial ovarian cancer tissues is correlated with parameters such as a high pathological grade and the spread of cancer cells to the lymph nodes, indicating that PARPi may play a role in the advancement of ovarian cancer (35). But PARP-1 is overexpressed and may also stimulate angiogenesis in epithelial ovarian cancer cells by increasing the expression of VEGFA (35). Preclinical studies have demonstrated that the combination of PARPi and Ais exhibits a synergistic effect, effectively inhibiting the invasion of ovarian cancer cells and the creation of microvascular endothelial tubes (29, 36). PARPi and Ais modify the genetic makeup of tumor cells, both directly and indirectly, in order to enhance the effectiveness of therapy (36). Furthermore, PARP inhibitors could be used in combination with other targeted therapies, including immunotherapies such as PD-1/PD-L1 inhibitors, to enhance the ability to attack tumor cells (37). However, the precise mechanisms underpinning these combinations remain poorly understood and may vary depending on the specific anti-angiogenic drugs involved. Additional research is required to clarify the precise mechanism through which this combination produces its anticancer effects.

With respect to safety, the rate of Grade≥ 3 TRAEs was determined to be 22%, indicating that the occurrence of negative effects from the combination therapy was deemed acceptable. In clinical trials examining the use of Ais alone, the most often seen Grade≥ 3 TRAEs were high blood pressure, blood clotting events, low levels of neutrophils, and bleeding outside of the central nervous system (35, 36). In clinical trials examining the use of PARPi alone, the most often seen Grade≥ 3 TRAEs were anemia, thrombocytopenia, neutropenia, fatigue, and nausea (38–40). Our results showed that the most often seen Grade≥ 3 TRAEs of the combined medication were stomach pain, hypertension, and anemia, which align with the adverse reactions typically associated with monotherapy.

The present study had numerous strengths. First, our study was an updated systematic review and meta-analysis to evaluate the effectiveness and safety of PARPi in conjunction with Ais for advanced ovarian cancer. Though Wei Y et al. conducted a meta-analysis about effectiveness and safety of PARPi plus Ais for advanced ovarian cancer, there were serious drawbacks in the previous meta-analysis (34). Four of the seven studies included in the previous meta-analysis contained duplicate patients from the PAOLA-1 trials, which led to a significant bias (34). Our study performed an expanded search strategy with additional search terms to ensure a comprehensive literature review, and nine trials studying PARPi plus Ais for advanced ovarian cancer were included, which resulted in more accurate results. Additionally, the IPDformKM package was utilized to reconstruct Kaplan-Meier curves for PFS and OS, providing a clear and comprehensible representation of oncological outcomes. Furthermore, a subgroup analysis was performed to resolve discrepancies in the patient inclusion criteria observed in different studies, which encompassed platinum-resistant recurrent ovarian cancer, platinum-sensitive recurrent ovarian cancer, and newly diagnosed advanced ovarian cancer. This study provides empirical support to inform the practical application of Ais in combination with PARPi for the management of patients with advanced ovarian cancer.

Undoubtedly, our study has specific limitations. First, the sample size was quite small. The analysis included only nine trials, involving a total of 1074 individuals diagnosed with advanced ovarian cancer who were treated with Ais plus PARPi. Second, the inclusion of single-arm clinical trials resulted in indirect comparisons between different treatment protocols. We failed to perform the meta-analysis of HR value estimates from Kaplan–Meier curves regarding PFS or OS. One reason was that several single-arm trials did not provide HR value since there was not a controlled group. Another reason was that the regimens of the control groups in different RCTs were not the same. As an alternative method, Kaplan-Meier curves for OS and PFS were reconstructed using the IPDformKM package, which presented an intuitive representation for oncological outcomes. This method has been reported previously by Guyot et al. (14). Third, the nine studies exhibited significant variation in terms of research technique, patient characteristics, and treatment regimens. Therefore, the explanation of our findings requires a certain level of caution.

In conclusion, the combination of PARPi and Ais is both viable and safe for treating advanced ovarian cancer. The findings of our study validate the recommendation of combination therapy as the primary treatment option for individuals diagnosed with advanced ovarian cancer. Our findings show improved survival and progression-free survival, as well as a reduction in treatment-related adverse events, in platinum-resistant recurrent ovarian cancer and in newly diagnosed advanced ovarian cancer. However, these results need to be further validated in a larger sample size and with longer follow-up.

## Data Availability

The datasets presented in this study can be found in online repositories. The names of the repository/repositories and accession number(s) can be found in the article/**Supplementary Material**.
