# Supplementary material for: Efficacy and safety of angiogenesis inhibitors combined with poly ADP ribose polymerase inhibitors in the maintenance treatment of advanced ovarian cancer: a meta-analysis
Source: Front Oncol. 2024 Nov 18;14:1477105. doi: 10.3389/fonc.2024.1477105 (PMC11609078; doi:10.3389/fonc.2024.1477105)
Supplement: Supplementary file 1 [file DataSheet1.docx]

Pubmed

| #1 | (((((((((((((((((Ovarian Neoplasms[Title/Abstract]) OR (Ovary Cancers[Title/Abstract])) OR (Ovarian Cancer[Title/Abstract])) OR (Cancer of Ovary[Title/Abstract])) OR (Cancer of the Ovary[Title/Abstract])) OR (Cancer, Ovarian[Title/Abstract])) OR (Cancers, Ovarian[Title/Abstract])) OR (Cancers, Ovary[Title/Abstract])) OR (Ovarian Cancers[Title/Abstract])) OR (Ovary Cancer[Title/Abstract])) OR (Cancer, Ovary[Title/Abstract])) OR (Neoplasm, Ovary[Title/Abstract])) OR (Neoplasm, Ovarian[Title/Abstract])) OR (Ovary Neoplasm[Title/Abstract])) OR (Ovarian Neoplasm[Title/Abstract])) OR (Neoplasms, Ovarian[Title/Abstract])) OR (Neoplasms, Ovary[Title/Abstract])) OR (Ovary Neoplasms[Title/Abstract]) | 75893 |
| --- | --- | --- |
| #2 | ((((((((((((((((((((poly-ADP-ribose polymerase[Title/Abstract]) OR (poly (adp ribose) polymerase[Title/Abstract])) OR (poly (ADP-ribose) polymerase-1[Title/Abstract])) OR (poly ADP ribose polymerase[Title/Abstract])) OR (poly ADP ribose polymerase 1[Title/Abstract])) OR (poly ADP ribose polymerase 2[Title/Abstract])) OR (poly ADP ribose polymerase inhibitor[Title/Abstract])) OR (poly (adp ribose) polymerase[Title/Abstract])) OR (poly (ADP ribose) polymerase 1[Title/Abstract])) OR (poly(ADP ribose) polymerase 2[Title/Abstract])) OR (poly (ADP ribose) polymerase inhibitor[Title/Abstract])) OR (poly (ADP ribose) polymerase inhibitors[Title/Abstract])) OR (poly (adp ribose) polymerase[Title/Abstract])) OR (poly (ADP-ribose) polymerase inhibitors[Title/Abstract])) OR (poly-ADP-ribose polymerase[Title/Abstract])) OR (niraparib[Title/Abstract])) OR (olaparib[Title/Abstract])) OR (veliparib[Title/Abstract])) OR (rucaparib[Title/Abstract])) OR (talazoparib[Title/Abstract])) OR (fluzoparib[Title/Abstract]) | 19891 |
| #3 | ((((((((((((((((((((((((((((((((((((((((((((((((((((((((((((((((((((((((Angiogenesis Inhibitors[Title/Abstract]) OR (Effect, Anti-Angiogenesis[Title/Abstract])) OR (Anti Angiogenesis Effects[Title/Abstract])) OR (Effect, Antiangiogenesis[Title/Abstract])) OR (Anti Angiogenesis Effect[Title/Abstract])) OR (Anti-Angiogenesis Effects[Title/Abstract])) OR (Antiangiogenesis Effect[Title/Abstract])) OR (Antiangiogenesis Effects[Title/Abstract])) OR (Anti-Angiogenesis Effect[Title/Abstract])) OR (Effects, Anti-Angiogenesis[Title/Abstract])) OR (Effects, Antiangiogenesis[Title/Abstract])) OR (Inhibitor, Angiogenesis Factor[Title/Abstract])) OR (Factor Inhibitor, Angiogenesis[Title/Abstract])) OR (Factor Inhibitors, Angiogenesis[Title/Abstract])) OR (Angiogenesis Factor Inhibitors[Title/Abstract])) OR (Inhibitors, Angiogenesis Factor[Title/Abstract])) OR (Angiogenesis Factor Inhibitor[Title/Abstract])) OR (Neovascularization Inhibitors[Title/Abstract])) OR (Antagonists, Angiogenic[Title/Abstract])) OR (Antiangiogenic Agents[Title/Abstract])) OR (Inhibitor, Neovascularization[Title/Abstract])) OR (Agents, Anti-Angiogenetic[Title/Abstract])) OR (Angiogenic Antagonists[Title/Abstract])) OR (Angiogenetic Inhibitors[Title/Abstract])) OR (Drug, Anti-Angiogenic[Title/Abstract])) OR (Inhibitors, Neovascularization[Title/Abstract])) OR (Anti-Angiogenic Drugs[Title/Abstract])) OR (Anti Angiogenic Drug[Title/Abstract])) OR (Anti Angiogenetic Agents[Title/Abstract])) OR (Antagonist, Angiogenetic[Title/Abstract])) OR (Inhibitors, Angiogenic[Title/Abstract])) OR (Antagonist, Angiogenic[Title/Abstract])) OR (Antagonists, Angiogenetic[Title/Abstract])) OR (Neovascularization Inhibitor[Title/Abstract])) OR (Agent, Angiostatic[Title/Abstract])) OR (Angiogenic Antagonist[Title/Abstract])) OR (Angiogenic Inhibitor[Title/Abstract])) OR (Angiostatic Agent[Title/Abstract])) OR (Antiangiogenic Agent[Title/Abstract])) OR (Anti Angiogenic Drugs[Title/Abstract])) OR (Angiostatic Agents[Title/Abstract])) OR (Agents, Antiangiogenic[Title/Abstract])) OR (Anti-Angiogenetic Agents[Title/Abstract])) OR (Inhibitor, Angiogenetic[Title/Abstract])) OR (Drugs, Anti-Angiogenic[Title/Abstract])) OR (Anti Angiogenetic Agent[Title/Abstract])) OR (Angiogenesis Inhibitor[Title/Abstract])) OR (Inhibitors, Angiogenetic[Title/Abstract])) OR (Inhibitor, Angiogenic[Title/Abstract])) OR (Angiogenetic Antagonists[Title/Abstract])) OR (Angiogenetic Inhibitor[Title/Abstract])) OR (Agent, Antiangiogenic[Title/Abstract])) OR (Inhibitor, Angiogenesis[Title/Abstract])) OR (Angiogenic Inhibitors[Title/Abstract])) OR (Agent, Anti-Angiogenetic[Title/Abstract])) OR (Angiogenetic Antagonist[Title/Abstract])) OR (Anti-Angiogenic Drug[Title/Abstract])) OR (Inhibitors, Angiogenesis[Title/Abstract])) OR (Anti-Angiogenetic Agent[Title/Abstract])) OR (Agents, Angiostatic[Title/Abstract])) OR (cediranib[Title/Abstract])) OR (bevacizumab[Title/Abstract])) OR (sorafenib[Title/Abstract])) OR (apatinib[Title/Abstract])) OR (aflibercept[Title/Abstract])) OR (ramucirumab[Title/Abstract])) OR (axitinib[Title/Abstract])) OR (cabozantinib[Title/Abstract])) OR (lenvatinib[Title/Abstract])) OR (pazopanib[Title/Abstract])) OR (regorafenib[Title/Abstract])) OR (sunitinib[Title/Abstract])) OR (vandetanib[Title/Abstract]) | 72177 |
| #4 | (((((((((((Randomized Controlled Trial[Title/Abstract]) OR (Controlled clinical tria[Title/Abstract])) OR (random allocation[Title/Abstract])) OR (double-blind[Title/Abstract])) OR (single-blind[Title/Abstract])) OR (Placebo[Title/Abstract])) OR (Randomly[Title/Abstract])) OR (Randomized[Title/Abstract])) OR (clinical trial[Title/Abstract])) OR (Trial[Title/Abstract])) OR (RCT[Title/Abstract])) OR (random[Title/Abstract]) | 1874008 |
| #5 | #1 and #2 and #3 and #4 | 117 |

Cochrane

| #1 | (Ovarian Neoplasms or Ovary Cancers or Ovarian Cancer or Cancer of Ovary or Cancer of the Ovary or Cancer, Ovarian or Cancers, Ovarian or Cancers, Ovary or Ovarian Cancers or Ovary Cancer or Cancer, Ovary or Neoplasm, Ovary or Neoplasm, Ovarian or Ovary Neoplasm or Ovarian Neoplasm or Neoplasms, Ovarian or Neoplasms, Ovary or Ovary Neoplasms):ab,ti,kw | 9508 |
| --- | --- | --- |
| #2 | ((poly-ADP-ribose polymerase or poly (adp ribose) polymerase or poly (ADP-ribose) polymerase-1 or poly ADP ribose polymerase or poly ADP ribose polymerase 1 or poly ADP ribose polymerase 2 or poly ADP ribose polymerase inhibitor or poly (adp ribose) polymerase or poly (ADP ribose) polymerase 1 or poly(ADP ribose) polymerase 2 or poly (ADP ribose) polymerase inhibitor or poly (ADP ribose) polymerase inhibitors or poly (adp ribose) polymerase or poly (ADP-ribose) polymerase inhibitors or poly-ADP-ribose polymerase or niraparib or olaparib or veliparib or rucaparib or talazoparib or fluzoparib)):ab,ti,kw | 1778 |
| #3 | (Angiogenesis Inhibitors or Effect, Anti-Angiogenesis or Anti Angiogenesis Effects or Effect, Antiangiogenesis or Anti Angiogenesis Effect or Anti-Angiogenesis Effects or Antiangiogenesis Effect or Antiangiogenesis Effects or Anti-Angiogenesis Effect or Effects, Anti-Angiogenesis or Effects, Antiangiogenesis or Inhibitor, Angiogenesis Factor or Factor Inhibitor, Angiogenesis or Factor Inhibitors, Angiogenesis or Angiogenesis Factor Inhibitors or Inhibitors, Angiogenesis Factor or Angiogenesis Factor Inhibitor or Neovascularization Inhibitors or Antagonists, Angiogenic or Antiangiogenic Agents or Inhibitor, Neovascularization or Agents, Anti-Angiogenetic or Angiogenic Antagonists or Angiogenetic Inhibitors or Drug, Anti-Angiogenic or Inhibitors, Neovascularization or Anti-Angiogenic Drugs or Anti Angiogenic Drug or Anti Angiogenetic Agents or Antagonist, Angiogenetic or Inhibitors, Angiogenic or Antagonist, Angiogenic or Antagonists, Angiogenetic or Neovascularization Inhibitor or Agent, Angiostatic or Angiogenic Antagonist or Angiogenic Inhibitor or Angiostatic Agent or Antiangiogenic Agent or Anti Angiogenic Drugs or Angiostatic Agents or Agents, Antiangiogenic or Anti-Angiogenetic Agents or Inhibitor, Angiogenetic or Drugs, Anti-Angiogenic or Anti Angiogenetic Agent or Angiogenesis Inhibitor or Inhibitors, Angiogenetic or Inhibitor, Angiogenic or Angiogenetic Antagonists or Angiogenetic Inhibitor or Agent, Antiangiogenic or Inhibitor, Angiogenesis or Angiogenic Inhibitors or Agent, Anti-Angiogenetic or Angiogenetic Antagonist or Anti-Angiogenic Drug or Inhibitors, Angiogenesis or Anti-Angiogenetic Agent or Agents, Angiostatic or cediranib or bevacizumab or sorafenib or apatinib or aflibercept or ramucirumab or axitinib or cabozantinib or lenvatinib or pazopanib or regorafenib or sunitinib or vandetanib):ab,ti,kw | 16417 |
| #4 | (Randomized Controlled Trial OR Controlled clinical tria OR random allocation OR double-blind OR single-blind OR Placebo OR Randomly OR Randomized OR clinical trial OR Trial OR RCT OR random):ab,ti,kw | 1516483 |
| #5 | #1and#2and#3and#4 | 217 |

Embase

| #1 | ‘Ovarian Neoplasms’:ab,ti,kw or ’Ovary Cancers’:ab,ti,kw or ’Ovarian Cancer’:ab,ti,kw or ’Cancer of Ovary’:ab,ti,kw or ’Cancer of the Ovary’:ab,ti,kw or ’Cancer, Ovarian’:ab,ti,kw or ’Cancers, Ovarian’:ab,ti,kw or ’Cancers, Ovary’:ab,ti,kw or ’Ovarian Cancers’:ab,ti,kw or ’Ovary Cancer’:ab,ti,kw or ’Cancer, Ovary’:ab,ti,kw or ’Neoplasm, Ovary’:ab,ti,kw or ’Neoplasm, Ovarian’:ab,ti,kw or ’Ovary Neoplasm’:ab,ti,kw or ’Ovarian Neoplasm’:ab,ti,kw or ’Neoplasms, Ovarian’:ab,ti,kw or ’Neoplasms, Ovary’:ab,ti,kw or ’Ovary Neoplasms’:ab,ti,kw | 112146 |
| --- | --- | --- |
| #2 | ‘poly-ADP-ribose polymerase’:ab,ti,kw or ‘poly (adp ribose) polymerase’:ab,ti,kw or ‘poly (ADP-ribose) polymerase-1’:ab,ti,kw or ‘poly ADP ribose polymerase’:ab,ti,kw or ‘poly ADP ribose polymerase 1’:ab,ti,kw or ‘poly ADP ribose polymerase 2’:ab,ti,kw or ‘poly ADP ribose polymerase inhibitor’:ab,ti,kw or ‘poly (adp ribose) polymerase’:ab,ti,kw or ‘poly (ADP ribose) polymerase 1’:ab,ti,kw or ‘poly(ADP ribose) polymerase 2’:ab,ti,kw or ‘poly (ADP ribose) polymerase inhibitor’:ab,ti,kw or ‘poly (ADP ribose) polymerase inhibitors’:ab,ti,kw or ‘poly (adp ribose) polymerase’:ab,ti,kw or ‘poly (ADP-ribose) polymerase inhibitors’:ab,ti,kw or ‘poly-ADP-ribose polymerase’:ab,ti,kw or ‘niraparib’:ab,ti,kw or ‘olaparib’:ab,ti,kw or ‘veliparib’:ab,ti,kw or ‘rucaparib’:ab,ti,kw or ‘talazoparib’:ab,ti,kw or ‘fluzoparib’:ab,ti,kw | 25355 |
| #3 | ‘Angiogenesis Inhibitors’:ab,ti,kw or ‘Effect, Anti-Angiogenesis’:ab,ti,kw or ‘Anti Angiogenesis Effects’:ab,ti,kw or ‘Effect, Antiangiogenesis’:ab,ti,kw or ‘Anti Angiogenesis Effect’:ab,ti,kw or ‘Anti-Angiogenesis Effects’:ab,ti,kw or ‘Antiangiogenesis Effect’:ab,ti,kw or ‘Antiangiogenesis Effects’:ab,ti,kw or ‘Anti-Angiogenesis Effect’:ab,ti,kw or ‘Effects, Anti-Angiogenesis’:ab,ti,kw or ‘Effects, Antiangiogenesis’:ab,ti,kw or ‘Inhibitor, Angiogenesis Factor’:ab,ti,kw or ‘Factor Inhibitor, Angiogenesis’:ab,ti,kw or ‘Factor Inhibitors, Angiogenesis’:ab,ti,kw or ‘Angiogenesis Factor Inhibitors’:ab,ti,kw or ‘Inhibitors, Angiogenesis Factor’:ab,ti,kw or ‘Angiogenesis Factor Inhibitor’:ab,ti,kw or ‘Neovascularization Inhibitors’:ab,ti,kw or ‘Antagonists, Angiogenic’:ab,ti,kw or ‘Antiangiogenic Agents’:ab,ti,kw or ‘Inhibitor, Neovascularization’:ab,ti,kw or ‘Agents, Anti-Angiogenetic’:ab,ti,kw or ‘Angiogenic Antagonists’:ab,ti,kw or ‘Angiogenetic Inhibitors’:ab,ti,kw or ‘Drug, Anti-Angiogenic’:ab,ti,kw or ‘Inhibitors, Neovascularization’:ab,ti,kw or ‘Anti-Angiogenic Drugs’:ab,ti,kw or ‘Anti Angiogenic Drug’:ab,ti,kw or ‘Anti Angiogenetic Agents’:ab,ti,kw or ‘Antagonist, Angiogenetic’:ab,ti,kw or ‘Inhibitors, Angiogenic’:ab,ti,kw or ‘Antagonist, Angiogenic’:ab,ti,kw or ‘Antagonists, Angiogenetic’:ab,ti,kw or ‘Neovascularization Inhibitor’:ab,ti,kw or ‘Agent, Angiostatic’:ab,ti,kw or ‘Angiogenic Antagonist’:ab,ti,kw or ‘Angiogenic Inhibitor’:ab,ti,kw or ‘Angiostatic Agent’:ab,ti,kw or ‘Antiangiogenic Agent’:ab,ti,kw or ‘Anti Angiogenic Drugs’:ab,ti,kw or ‘Angiostatic Agents’:ab,ti,kw or ‘Agents, Antiangiogenic’:ab,ti,kw or ‘Anti-Angiogenetic Agents’:ab,ti,kw or ‘Inhibitor, Angiogenetic’:ab,ti,kw or ‘Drugs, Anti-Angiogenic’:ab,ti,kw or ‘Anti Angiogenetic Agent’:ab,ti,kw or ‘Angiogenesis Inhibitor’:ab,ti,kw or ‘Inhibitors, Angiogenetic’:ab,ti,kw or ‘Inhibitor, Angiogenic’:ab,ti,kw or ‘Angiogenetic Antagonists’:ab,ti,kw or ‘Angiogenetic Inhibitor’:ab,ti,kw or ‘Agent, Antiangiogenic’:ab,ti,kw or ‘Inhibitor, Angiogenesis’:ab,ti,kw or ‘Angiogenic Inhibitors’:ab,ti,kw or ‘Agent, Anti-Angiogenetic’:ab,ti,kw or ‘Angiogenetic Antagonist’:ab,ti,kw or ‘Anti-Angiogenic Drug’:ab,ti,kw or ‘Inhibitors, Angiogenesis’:ab,ti,kw or ‘Anti-Angiogenetic Agent’:ab,ti,kw or ‘Agents, Angiostatic’:ab,ti,kw or ‘cediranib’:ab,ti,kw or ‘bevacizumab’:ab,ti,kw or ‘sorafenib’:ab,ti,kw or ‘apatinib’:ab,ti,kw or ‘aflibercept’:ab,ti,kw or ‘ramucirumab’:ab,ti,kw or ‘axitinib’:ab,ti,kw or ‘cabozantinib’:ab,ti,kw or ‘lenvatinib’:ab,ti,kw or ‘pazopanib’:ab,ti,kw or ‘regorafenib’:ab,ti,kw or ‘sunitinib’:ab,ti,kw or ‘vandetanib’:ab,ti,kw | 92055 |
| #4 | ‘Randomized Controlled Trial’:ab,ti,kw or ‘Controlled clinical tria’:ab,ti,kw or ‘random allocation’:ab,ti,kw or ‘double-blind’:ab,ti,kw or ‘single-blind’:ab,ti,kw or ‘Placebo’:ab,ti,kw or ‘Randomly’:ab,ti,kw or ‘Randomized’:ab,ti,kw or ‘clinical trial’:ab,ti,kw or ‘Trial’:ab,ti,kw or ‘RCT’:ab,ti,kw or ‘random’:ab,ti,kw | 2613105 |
| #5 | #1 and #2 and #3 and #4 | 304 |

Web of science

| #1 | TS=(Ovarian Neoplasms or Ovary Cancers or Ovarian Cancer or Cancer of Ovary or Cancer of the Ovary or Cancer, Ovarian or Cancers, Ovarian or Cancers, Ovary or Ovarian Cancers or Ovary Cancer or Cancer, Ovary or Neoplasm, Ovary or Neoplasm, Ovarian or Ovary Neoplasm or Ovarian Neoplasm or Neoplasms, Ovarian or Neoplasms, Ovary or Ovary Neoplasms) | 309321 |
| --- | --- | --- |
| #2 | TS=(poly-ADP-ribose polymerase or poly (adp ribose) polymerase or poly (ADP-ribose) polymerase-1 or poly ADP ribose polymerase or poly ADP ribose polymerase 1 or poly ADP ribose polymerase 2 or poly ADP ribose polymerase inhibitor or poly (adp ribose) polymerase or poly (ADP ribose) polymerase 1 or poly(ADP ribose) polymerase 2 or poly (ADP ribose) polymerase inhibitor or poly (ADP ribose) polymerase inhibitors or poly (adp ribose) polymerase or poly (ADP-ribose) polymerase inhibitors or poly-ADP-ribose polymerase or niraparib or olaparib or veliparib or rucaparib or talazoparib or fluzoparib) | 37502 |
| #3 | TS=(Angiogenesis Inhibitors or Effect, Anti-Angiogenesis or Anti Angiogenesis Effects or Effect, Antiangiogenesis or Anti Angiogenesis Effect or Anti-Angiogenesis Effects or Antiangiogenesis Effect or Antiangiogenesis Effects or Anti-Angiogenesis Effect or Effects, Anti-Angiogenesis or Effects, Antiangiogenesis or Inhibitor, Angiogenesis Factor or Factor Inhibitor, Angiogenesis or Factor Inhibitors, Angiogenesis or Angiogenesis Factor Inhibitors or Inhibitors, Angiogenesis Factor or Angiogenesis Factor Inhibitor or Neovascularization Inhibitors or Antagonists, Angiogenic or Antiangiogenic Agents or Inhibitor, Neovascularization or Agents, Anti-Angiogenetic or Angiogenic Antagonists or Angiogenetic Inhibitors or Drug, Anti-Angiogenic or Inhibitors, Neovascularization or Anti-Angiogenic Drugs or Anti Angiogenic Drug or Anti Angiogenetic Agents or Antagonist, Angiogenetic or Inhibitors, Angiogenic or Antagonist, Angiogenic or Antagonists, Angiogenetic or Neovascularization Inhibitor or Agent, Angiostatic or Angiogenic Antagonist or Angiogenic Inhibitor or Angiostatic Agent or Antiangiogenic Agent or Anti Angiogenic Drugs or Angiostatic Agents or Agents, Antiangiogenic or Anti-Angiogenetic Agents or Inhibitor, Angiogenetic or Drugs, Anti-Angiogenic or Anti Angiogenetic Agent or Angiogenesis Inhibitor or Inhibitors, Angiogenetic or Inhibitor, Angiogenic or Angiogenetic Antagonists or Angiogenetic Inhibitor or Agent, Antiangiogenic or Inhibitor, Angiogenesis or Angiogenic Inhibitors or Agent, Anti-Angiogenetic or Angiogenetic Antagonist or Anti-Angiogenic Drug or Inhibitors, Angiogenesis or Anti-Angiogenetic Agent or Agents, Angiostatic or cediranib or bevacizumab or sorafenib or apatinib or aflibercept or ramucirumab or axitinib or cabozantinib or lenvatinib or pazopanib or regorafenib or sunitinib or vandetanib) | 205422 |
| #4 | TS=(Randomized Controlled Trial OR Controlled clinical tria OR random allocation OR double-blind OR single-blind OR Placebo OR Randomly OR Randomized OR clinical trial OR Trial OR RCT OR random) | 5686510 |
| #5 | #1 and #2 and #3 and #4 | 583 |

Pubmed 117

Cochrane 217

Embase 304

Web of science 583
